# *In Vitro* and *In Vivo Antiproliferative Actions of Solanum gilo* Raddi (Solanaceae) Fruit Extract on Breast Tissues

**DOI:** 10.1155/2022/6834626

**Published:** 2022-09-26

**Authors:** Edwige Nana Tchoupang, Vincent Yadji, Jean Baptiste Wangbara, Marius Trésor Kemegne Sipping, Stéphane Zingue, Dieudonné Njamen

**Affiliations:** ^1^Department of Animal Science, Faculty of Agriculture and Veterinary Medicine, University of Buea, P.O. Box 63, Buea, Cameroon; ^2^Department of Life and Earth Sciences, Higher Teachers' Training College, University of Maroua, P.O. Box 55, Maroua, Cameroon; ^3^Department of Biochemistry, Faculty of Science, University of Yaounde 1, P.O. Box 812, Yaounde, Cameroon; ^4^Department of Medical and Biomedical Engineering, Higher Technical Teachers' Training College, University of Ebolowa, P.O. Box 886, Ebolowa, Cameroon; ^5^Department of Animal Biology and Physiology, Faculty of Science, University of Yaoundé 1, P.O. Box 812, Yaounde, Cameroon

## Abstract

**Background:**

Menopause is a normal event characterized by a drop in estrogen's production, leading to numerous symptoms. To face these later, women rely on hormone replacement therapy (HRT), which alleviates numerous menopausal symptoms. Unfortunately, long-term exposure to estrogens is associated with an increase in endometrial and breast cancers. This study dealt with the evaluation of *in vitro* and *in vivo* antiproliferative effects of *Solanum gilo* Raddi, a plant used in folk medicine to treat tumors in Cameroon.

**Materials and Methods:**

The *in vitro* antiproliferative effect of *S. gilo* fruit extract was investigated through the well-characterized MTT assay in one normal and three cancerous breast cells. For the *in vivo* study, one normal group (NOR) of rats received distilled water (vehicle), and five other groups (*n* = 6) were treated either with tamoxifen (3.3 mg/kg BW) as standard or with the vehicle (negative control) or *S. gilo* fruit hydroethanolic extract (125, 250, and 500 mg/kg BW). The treatments were administered concomitantly with the E2V to induce breast hyperplasia for 16 weeks, and the endpoints were the histopathology of the mammary glands and some biochemical parameters.

**Results:**

The *S. gilo* extract significantly inhibited human (MCF-7 and MDA-MB-231) and rodent (4T1) breast carcinoma cell growth. Rats exposed only to E2V presented atypical mammary hyperplasia compared to the normal parenchyma observed in normal rats. While rats treated with *S. gilo* extract at the dose of 125 mg/kg BW showed a microarchitecture of mammary glands with moderate hyperplasia, the higher doses (250 and 500 mg/kg) inhibited mammary gland hyperplasia compared to the E2V group.

**Conclusion:**

*S. gilo* fruit extract has antiproliferative constituents that could help to fight against estrogen-dependent breast cancer, thanks to their ability to scavenge free radicals, as exhibited in this study.

## 1. Introduction

Menopause is a genetically programmed event characterized by a drop in estrogen's production, leading to numerous physiological disorders including hot flushes, sleep disturbances, vaginal dryness, and changes in mood [1]. To overcome these symptoms, postmenopausal women often refer to hormone replacement therapy (HRT), which significantly reduces the frequencies of hot flushes and alleviates vaginal dryness and bone density in the short and medium term [2]. Unfortunately, the long-term use of HRT increases the risk of endometrial and breast cancers. Cancer is an important cause of morbidity and mortality worldwide, irrespective of the level of human development. In women, breast cancer is the most frequently diagnosed cancer and the leading cause of cancer-associated death worldwide. Although hereditary and genetic factors account for 5% to 10% of breast cancer cases, factors linked to estrogen exposure are the major drivers of the observed global incidence [3]. Some of these risk factors are related to menstruation (early menarche, later menopause), reproduction (nulliparity, first birth at late age, and fewer children), and exogenous hormone intake (oral contraceptives and hormone replacement therapy, HRT) [3–5]. Recent studies have reported a decline in breast cancer mortality and daily rates in developed countries and an increase in developing countries because of differences in accessibility to novel drugs and the application of clinical guidelines [6].

Therapeutic management of breast cancer includes surgery, radiotherapy, chemotherapy, immunotherapy, and hormonotherapy (e.g., tamoxifen) [7, 8]. However, in spite of the substantial progress in the treatment of cancer, its high cost, late diagnosis, side effects, and poor health services especially in Africa are limiting factors [9]. Besides, resistance to medications and their high toxicity poses a great problem [10]. The primary prevention of breast cancer could be through the use of traditional medicine [11]. Indeed, epidemiological and preclinical studies have shown that the consumption of natural food fibers or plant compounds can prevent the development of cancer [12, 13]. In the same line with that, *Solanum gilo* Raddi (Solanaceae) is a plant, that is, consumed in Cameroon. Some of the phytoconstituents of the fruit extract of *Solanum gilo* include; large quantities of vitamin C, flavonoïds, and saponins which have powerful antioxidants, anti-inflammatory, and anticancer properties [14–16]. However, to the best of our knowledge, there is no study on the antiproliferative properties of *S. gilo* fruits. Therefore, this study was designed to evaluate the cytotoxicity of the hydroethanolic extract of *Solanum gilo* fruits in human (MCF-7 and MDA-MB-231) and rodent (4T1) breast carcinoma cells growth as well as its *in vivo* protective effect against E_2_V-induced breast hyperplasia in female Wistar rats.

## 2. Materials and Methods

### 2.1. Chemicals, Buffer, and Medium

The salt 3-(4,5-dimethylthiazol-2-yl)-2,5-diphenyltetrazolium bromide (MTT) was from Roche Diagnostics (Penzberg, Germany). Antibiotics (penicillin and streptomycin), the HEPES buffer [2-[4-(2-hydroxyethyl) piperazin-1-yl]ethane sulfonic acid], GlutaMAX, and fetal bovine serum (FBS) were obtained from Gibco/Invitrogen (Karlsruhe, Germany). Estradiol valerate (Progynova®) and citrate of tamoxifen (Mylan®) were purchased from DELPHARM (Lille, France) and MYLAN SAS (Saint-Priest, France), respectively.

### 2.2. Plant Collection, Authentication, and Preparation of the Extract

*Solanum gilo* fruits were purchased from Pitoa market (Garoua, North Region of Cameroon) in April 2017, authenticated by Dr. Tchopsala (Botanist, Faculty of Science University of Maroua) and the voucher specimen (no. 14602 SFR/Cam) was deposited in the Cameroon National Herbarium (CNH). Two thousand grams (2,000 g) of fruit powder was soaked in hydroethanolic (v/v: 30/70) (6 L × 2, 48 h/extraction) at room temperature. After filtration (Whatman no. 4), ethanol was recovered under vacuum/reduced pressure (175 mbar) at 40°C using a rotary evaporator followed by lyophilization, and 20 g (1%) of the extract was obtained.

### 2.3. Phytochemical Screening

#### 2.3.1. Determination of Flavonoïd Content

The flavonoid content in the extract was estimated by aluminum chloride colorimetric method using 0.01% quercetin as standard [16]. To do so, 3 g of *S. gilo* extract in 100 mL of 70% ethanol was added to 2.5% aluminum chloride in methanol (2/1.2/6.8) while, AlCl was replaced by methanol in the blank. The mixture was incubated at room temperature for 45 minutes and the absorbance was measured at 430 nm using a Shimadzu UV-1700 spectrophotometer. The total flavonoid content was expressed as mg of quercetin per gram of extract of *S. gilo*. Each test was repeated three times.

#### 2.3.2. Determination of Flavonol Content

The method reported by Zhishen et al. [15] helps to estimate the flavonols content in *S. gilo* extract. Briefly, the standard or extract (0.1 mg/mL) was added to 2% AlCl_3_-ethanol and 50 g/L sodium acetate, incubated at 20°C for 2.5 h, and absorption was read at 440 nm through a Shimadzu UV-1700 spectrophotometer. Total flavonols content was expressed as mg of quercetin/g of dried extract. Each test was done thrice.

#### 2.3.3. Determination of Total Phenolic Content

The Folin–Ciocalteu method with gallic acid as standard [14] was used to estimate the total phenolic content in *S. gilo* extract. Folin–Ciocalteu reagent was added to standard or *S. gilo* extract at proportion 1/10 followed by the addition of Na_2_CO_3_ (20%) (1 : 2). The obtained mixture was then incubated for 30 min in the dark and absorbance was measured at 760 nm through a Shimadzu UV-1700 spectrophotometer.

#### 2.3.4. Determination of Total Protein Content

Total proteins in *S. gilo* extract were quantified using Bradford reagents [17] with bovine serum albumin (BSA) as standard. Equal volumes of *S. gilo* extract (1 mL) and freshly prepared Bradford reagent were mixed, incubated in the dark for 30 min, and absorbance was measured at 595 nm using a Shimadzu UV-1700 spectrophotometer.

### 2.4. In Vitro Antioxidant Screening

#### 2.4.1. DPPH (1,1-Diphenyl-2-picryl Hydrazyl) Assay

The scavenging of DPPH radicals of *S. gilo* extract was determined by their ability to donate hydrogen [17]. Indeed, 400 *μ*mol/L of DPPH in methanol was prepared just before use and 500 *μ*L of the solution was added to *S. gilo* extract (100–300 *μ*g/mL) or ferulic acid (positive control). The solution was incubated in the dark at 37°C for 30 min and absorbance was measured at 517 nm using a Shimadzu UV-1700 spectrophotometer. The DPPH radical scavenging effect or “inhibition percentage” was calculated: percentage of inhibition (%) = {[(A0−A1)/A0] × 100} where A0 is the absorbance of the control reaction and *A*1 is the absorbance of the sample.

#### 2.4.2. 2,2-Azino-bis-3-ethylbenzothiazoline-6-sulphonic Acid (ABTS) Assay

ABTS free radical scavenging activity was evaluated following the method described by Re et al. [18]. In summary, ABTS reagent (1 mL) was added to 100 *μ*L of *S. gilo* extract (100–300 *μ*g/mL) or ferulic acid (positive control). The mixture was incubated in the dark for 30 min and absorbance was measured at 734 nm using a Shimadzu UV-1700 spectrophotometer. The ABTS radical scavenging effect was calculated as stated in section 2.4.1 mentioned above.

### 2.5. In Vitro Cell Growth Assay

#### 2.5.1. Cell Lines

In this study, the following cancerous cell lines, acquired from the American Type Culture Collection (ATCC)/LGC Promochem (Wesel, Germany), were used: human ER-positive breast adenocarcinoma cells (MCF-7); human ER-negative breast adenocarcinoma cells (MDA-MB 231); human mammary epithelial cells (HMEC); and rodent breast adenocarcinoma cells (4T-1).

#### 2.5.2. Cell Culture

HMEC cells were cultured in Dulbecco's modified eagle medium (DMEM) supplemented with 10% fetal calf serum (FCS) while MCF-7, MDA-MB 231, and 4T-1 cells were cultured in RPMI 1640 medium supplemented with 10% fetal bovine serum (FBS). Both media were also supplemented with penicillin (100 U/mL), streptomycin (100 *μ*g/mL), and HEPES (10 mM). The cell cultures were maintained at 37^o^C, pH 7.4 in a 5% CO_2_ humidified atmosphere, and cell passaging was done every two days. Prior to each experiment, cells were stained with trypan blue, and viable cells were counted using a Neubauer chamber.

#### 2.5.3. Tumor Cell Growth with S. gilo Hydroethanolic Extract

Cell growth was evaluated using 3-(4.5-dimethylthiazol-2-yl)-2.5-diphenyltetrazolium bromide (MTT) dye reduction assay. Cancer cells (100 *μ*l, 1 × 10^4^ cells/mL) were seeded in 96-well microplates. *S. gilo* extracts dissolved in 0.01% DMSO (5 to 200 *μ*g/mL) or 0.01% DMSO (control) was added to the cells in the wells. The cells were incubated and after 24, 48, and 72 h, MTT (0.5 mg/mL) was added and further incubated for 4 h. Thereafter, cells were lysed in a buffer containing 10% SDS in 0.01 M HCl, incubated overnight and absorbance measured at 570 nm using an ELISA reader. Each test was done in triplicate and repeated thrice. After subtracting background absorbance, the mean of cell number is presented as the results.

### 2.6. In Vivo Experiment

#### 2.6.1. Animals

Healthy female Wistar rats (41–51 days old; 70–85 g) were acquired from the breeding facility of the Animal Physiology Laboratory, University of Yaounde 1 (Cameroon). The rats were housed in clean plastic cages at room temperature (around 25°C) and had free access to a standard soy-free rat diet and water. The composition of animal diet was: corn (36.7%), bone flour (14.5%), wheat (36.6%), fish flour (4.8%), crushed palm kernel (7.3%), sodium chloride (0.3%), and vitamin complex (Olivitazol®- 0.01%).

#### 2.6.2. Ethical Consideration

The housing of animals and all experiments were conducted in accordance with the principles of the Cameroon Institutional National Ethic Committee (CEE Council 86/609; Reg. no. FWA-IRD 0001954), which follows the recommendations of the European Union on the protection of experimental animals.

#### 2.6.3. Estradiol-Induced Breast Hyperplasia in Rats

Thirty-six female rats (41–51 days old) were acclimatized for 7 days and randomly allocated into 6 groups (*n* = 6). Group 1 (normal control, NOR); group 2 (negative control, E2V) received the vehicle (distilled water); and group 3 (positive control, TAMOX + E2V) was treated with tamoxifen 3.3 mg/kg BW. The remaining groups (groups 4, 5, and 6) were treated with the hydroethanolic extract of *Solanum gilo* fruits at 125, 250, and 500 mg/kg BW. Treatment was done by oral gavage for 7 days before breast hyperplasia was induced and continued until 4 months (112 days). The breast hyperplasia was induced in groups 2–6 by chronic administration of E2V (1 mg/kg BW) as reported by Mense et al. [19] while group 1 (normal control) received distilled water. Weighing of the rats was done weekly and palpation twice a week from the first day of acclimatization until the end of the experiment to check the development of mammary tumors. After treatment (112 days, all surviving rats were weighed and sacrificed by decapitation under light anesthesia (10 mg/kg BW diazepam and 50 mg/kg BW ketamine *i.p.*) after a 12 h overnight nonhydric fasting. Blood samples were collected in anticoagulant (EDTA) tubes and dry for hematological and biochemical assays, respectively. The blood collected in the dry tubes was centrifuged at 3000 × *g* for 15 min at 4°C. The skin was dissected to expose mammary glands and estrogen target organs (ovaries, uterus, mammary gland, and femur), and other organs (kidneys, brain, spleen, adrenals, lungs, and liver) were removed and weighed. Finally, the mammary glands, kidney, brain, lung, and liver were fixed in 10% buffered formalin for histomorphological analysis.

### 2.7. Histological Analysis

Organs (mammary gland, lung, kidney, liver, and brain) embedded in paraffin were cut into 5 *μ*m sections and stained with hematoxylin and eosin. Histoarchitectures of abovementioned organs were examined using Axioskop 40 microscope connected to a computer where the images were transferred using MRGrab1.0 and Axio Vision 3.1 softwares, all from Zeiss (Hallbergmoos, Germany).

### 2.8. Biochemical and Hematological Analysis

Reagent kits acquired from Fortress Diagnostics Limited (Muckamore, United Kingdom) were used to determine aspartate transaminase (AST) and alanine transaminase (ALT) activities.

Hematological parameters (white blood cell (WBC) count, lymphocytes, monocytes, granulocytes, red blood cell (RBC) count, hematocrit, hemoglobin, mean corpuscular volume (MCV), mean corpuscular hemoglobin (MCH), mean corpuscular hemoglobin concentration (MCHC), and platelets) were evaluated using a Mindray BC-2800 Auto Hematology Analyzer Shenzhen Mindray Bio-Medical Electronics Co., Ltd.

### 2.9. Statistical Analysis

For each experimental group, results are presented as means ± SEM. Statistical analysis of data was carried out using GraphPad Prism software version 5.03. One-way analysis of variance (ANOVA) followed by Dunnett's posthoc test for multiple comparisons was performed to determine differences between treatment groups. The statistically significant difference was considered at *p* < 0.05.

## 3. Results

### 3.1. Phytoconstituents in *S. gilo* Extract

Quantitative phytochemical analysis showed that the hydroethanolic extract of *S. gilo* contains; by gram of dried weight 81.93 ± 3.19 mg total proteins eq BSA, 26.79 ± 0.22 mg total phenols eq to quercetin, 15.94 ± 0.07 mg flavonoids eq to quercetin, 1.59 ± 0.06 mg flavonols eq to quercetin (Table 1). Moreover, the EC_50_ values of the extract were 245.6 ± 8.3 *μ*g/mL (DPPH) and 147.1 ± 4.9 *μ*g/mL (ABTS) (Table 1).

### 3.2. Effects of *S. gilo* Hydroethanolic Extract on Cell Growth

**Figure 1:**
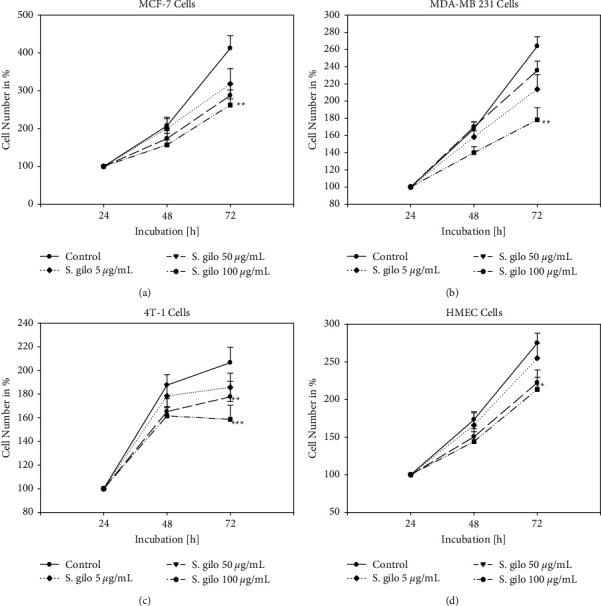
Growth of MCF-7 (a), MDA-MB 231 (b), 4T-1 (c), and HMEC (d) cells. Cells were treated with different concentrations of *Solanum gilo* hydroethanolic extract for 24, 48, and 72 hours. Controls remained untreated (*n* = 3). Treated tumor cell cultures were compared to nontreated control cultures of the same passage and cell numbers per well. ^*∗∗*^*p* < 0.05 and ^*∗∗*^*p* < 0.01 as compared to untreated control cells.

The ability of *S. gilo* extracts to inhibit the growth of MCF-7, MDA-MB 231, and 4T-1 cells was concentration-dependent. The magnitude of cytotoxic activity also increased proportionally with time (Figure 1). Only significant results at an optimum concentration of *S. gilo* (5, 50, and 100 *μ*g/mL) are presented. The effect on HMEC cells was less potent than those observed in tumoral cells, suggesting a selective action.

### 3.3. Effects of *S. gilo* Hydroethanolic Extract on E_2_V-Induced Hyperplasia in Rat

#### 3.3.1. Effects of *S. gilo* Extract on Body Weight

**Figure 2:**
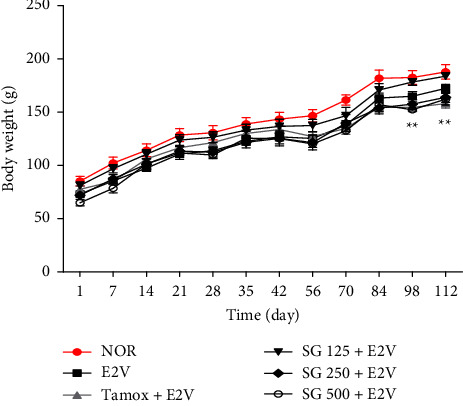
Body weight of rats after 16 weeks of treatment with different substances. NOR = Normal control treated with distilled water; E2V = E2V-control treated with distilled water; SG + E2V = Animals treated with the *S. gilo* hydroethanolic extract at the doses of 125, 250, and 500 mg/kg BW. TAMOX + E2V = Animals treated with tamoxifen (3.3 mg/kg BW); all groups except the normal group (NOR) received chronic administration of E2V at the dose of 1 mg/kg BW. Data are represented as mean ± SEM (*n* = 6). ^*∗∗*^*p* < 0.01 as compared to the negative control.

Among the experimental groups, there was no change in body weights. However, animals in the tamoxifen group had significantly (*p* < 0.01) lower body weights compared to the negative control group (E2V) from 98-days until the end of treatment (Figure 2).

#### 3.3.2. Histomorphological Analysis of the Mammary Glands

**Figure 3:**
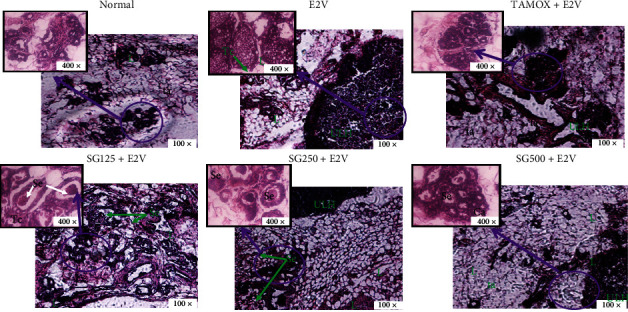
Effects of *S. gilo* hydroethanolic extract on microphotographs H&E 100× and 400× of mammary glands after 16 weeks of treatment. NOR = Normal control treated with distilled water; E2V = E2V-control treated with distilled water; SG + E2V = Animals treated with the *S. gilo* hydroethanolic extract at the doses of 125, 250, and 500 mg/kg BW. TAMOX + E2V = Animals treated with tamoxifen (3.3 mg/kg BW); all groups except the normal group (NOR) received chronic administration of E2V at the dose of 1 mg/kg BW. La = lumen of alveoli; At = adipose tissue; Se = eosinophil secretion, *L* = lobular; HLU = hyperplastic lobular unit.

Photomicrographs of mammary glands of animals from the normal control group showed normal acini surrounded by small amounts of connective tissue and significant amounts of adipose tissue (Figure 3). Meanwhile, animals that received chronic administration of E2V had high hyperplasia characterized by dilated ducts filled with atypical hyperplasia cells and reduced conjunctive tissue. However, the group treated with tamoxifen had quasinormal structures and no signs of hyperplasia. Animals treated with *S. gilo* hydroethanolic extract presented with hyperplasia (whose incidence was inversely proportional to the dose of extract) with low cellular proliferation and low ductal dilation (Figure 3).

#### 3.3.3. Effects on Organ Wet Weight

Table 2 shows the average organ wet weights after 16 weeks of treatment with *S. gilo* extract. There was a significant increment in lung (*p* < 0.05), spleen (*p* < 0.01), and brain relative wet weights compared to normal animals. In addition, animals treated with tamoxifen showed a significant (*p* < 0.001) decrease in ovarian and uterine wet weights compared to animals in the negative control group (E2V). There was a significant (*p* < 0.01) decrease in ovarian and spleen wet weights observed all tested doses of *S. gilo* extract compared to animals in the negative control group (E2V).

#### 3.3.4. Effects on Some Biochemical Parameters

There was a significant increase in AST activity in the negative control group (*p* < 0.05) compared to the normal rats. Moreover, animals that received *S. gilo* extract at 500 mg/kg BW presented a significant (*p* < 0.01) decrease in ALT activity compared to the negative control group (E2V) (Table 3).

Table 3 also shows the hematological profile of animals following 16 weeks of treatment. There was a significant (*p* < 0.01) decrease in the number of serum white blood cells in rats treated with *S. gilo* extract at the dose of 125 mg/kg BW compared to animals of the normal group. There was also a significant decrease in serum white blood cell count in SG 250 + E2V and SG 500 + E2V animals compared to the negative control group. Also, *S. gilo* extracts all tested doses caused a significant (*p* < 0.05) increase in monocyte levels compared to animals receiving only E2V. The monocyte level also increased significantly (*p* < 0.05) in the positive control group (TAMOX + E2V) compared to the normal control group. The platelet count also decreased significantly (*p* < 0.001) in the SG 250 + E2V group compared to the animals in the negative control group (E2V).

#### 3.3.5. Effects on Liver, Lung, Kidney, and Brain

**Figure 4:**
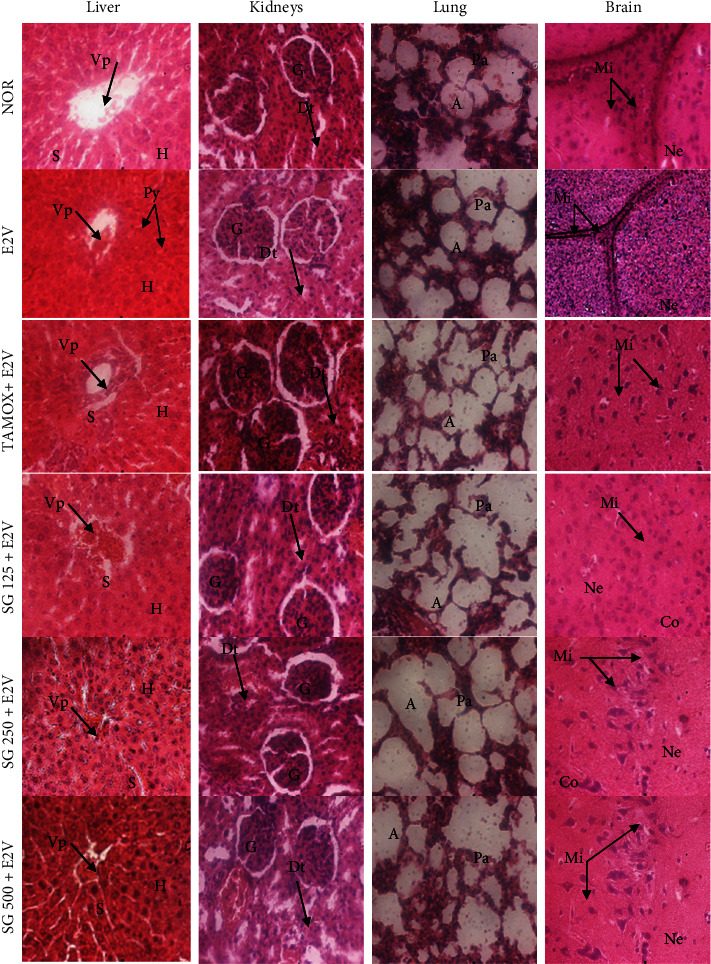
Effects of *S. gilo* hydroethanolic extract on microphotographs H and E 400× of liver, kidney, lung, and brain after 16 weeks of treatment. NOR = Normal control treated with distilled water; E2V = E2V-control treated with distilled water; SG + E2V = Animals treated with the *S. gilo* hydroethanolic extract at the doses of 125, 250, and 500 mg/kg BW. TAMOX + E2V = Animals treated with tamoxifen (3.3 mg/kg BW); all groups except the normal group (NOR) received chronic administration of E2V at the dose of 1 mg/kg BW. Data are represented as mean ± SEM (n = 6). Vp = Veine portal; H = Hepatocyte; S = Sinusoids; Py = Pyknotic nuclei; A = Alveoli; Ab = Aveolar bag; G = Glomerula; Dt = Distal tube; Pt = Proximal tube; Mi = Microglie; Ne = Neurone; Co = Cortex.

The major organs that can be exposed to toxic substances are the liver, lungs, kidneys, and brain. After 16 weeks of treatment, no changes were observed in the histo-architecture of the brain, lungs, and kidneys (Figure 4). Normal rats present normal parenchyma with mononuclear hepatocytes; kidneys with urinary space and well-differentiated glomerulus and their lungs were normal with normal alveolar bags. However, there was an increase in the number of pyknotic nuclei in the liver sections of animals treated chronically with E2V. These results are in line with the increase in liver wet weight observed in these animals. The animals treated with the extract had dose-dependent protection against E2V-induced liver injury with a better effect at the dose of 500 mg/kg BW.

## 4. Discussion

About 50,000 new cases of breast cancer are diagnosed in women each year with a very high mortality rate [3]. This mortality rate will remain high if there is no advancement in the production of safer medications [20]. Some medicinal plants have been reported to possess phytoconstituents with anticancer properties. Therefore, the *in vitro* and *in vivo* effects of the hydroethanolic extract from the fruits of *Solanum gilo* were evaluated in this study. Regarding *in vitro* antitumoral assay, it is noteworthy to recall that the MTT assay measures the ability of a substance to impact cell growth/viability by alteration of one or more cellular functions through the evaluation of cell protein production [21]. In line with our observed results, *S. gilo* hydroethanolic extract considerably decreased the growth of MCF-7, MDA-MB 231, and 4T-1 cells in a concentration-dependent manner. This antiproliferative effect of *S. gilo* extract is likely due to the presence of phytoconstituents with anticancer properties. Moreover, results from the preliminary phytochemical analysis showed that *S. gilo* hydroethanolic extract contains flavonoïds, particularly flavonols. Seal et al. [13] reported the presence of naringenin (flavanone), catechin (flavanol), and rutin in the fruits of *S. gilo*. These compounds are well-known antiproliferative agents. Indeed, naringenin was reported to induce cell death in epidermoid and hepatocellular carcinoma cells through the intrinsic pathway of apoptosis characterized by: ROS generation, mitochondrial depolarization, nuclear condensation, DNA fragmentation, cell cycle arrest in *G*_0_/*G*_1_ phase, and caspase-3 activation [22, 23]. These aforementioned mechanisms may explain the *in vitro* antiproliferative effects of *S. gilo* extract.

Mammary hyperplasia in rats was induced by oral administration of E2V (1 mg/mL BW) [19]. Mammary hyperplastic lobular units developed in animals treated with E2V after 16 weeks but normal rats had no mammary hyperplasia. Mense et al. [19] reported an atypical ductal proliferation in the mammary glands of rats treated with E2 for 120 days. Treatment with *S. gilo* hydroethanolic extract (all tested doses) or tamoxifen significantly reduced the mammary gland hyperplasia suggesting a protective effect of this extract. These results are supported by the ability of the constituents of this extract to inhibit human (MCF-7 and MDA-MB 231) as well as rodent (4T1) breast adenocarcinoma cells *in vitro*. Moreover, *S. gilo* extract contains anticancer compounds. Quercetin (the most abundant flavanol found in plants) and naringenin from citrus juice have been reported to inhibit breast carcinoma cell proliferation and delay mammary tumorigenesis induced in rodents [24]. The quasinormal architecture of breasts observed in animals treated with tamoxifen suggests that it prevented the development of breast hyperplasia, which is the step before estrogen-dependent cancers [25]. This result is in line with previous reports [26, 27]. Moreover, the results obtained after administration of the hydroethanolic extract of *S. gilo* suggest that this extract could protect against breast hyperplasia induced by long-term exposure to E2V.

According to toxicological profile results, the low weights observed in tamoxifen-treated animals could be due to the anorectic effects of tamoxifen. Indeed, tamoxifen increases the synthesis of an anorectic peptide; pro-opiomelanocortin (POMC) [28]. These results corroborate those previously observed by Zingue et al. [26] and Silihe et al. [27]; they showed that tamoxifen can reduce body weight by up to 22% in continuously treated animals. There was also a substantial (*p* < 0.05) decrease in lung, liver, and adrenal wet weights in animals receiving 500 mg/kg BW *S. gilo* compared to the negative control group. The increase in uterine mass observed in the tamoxifen group is consistent with other studies that have shown that under the influence of estrogens and estrogenic compounds, endometrial cells proliferate and differentiate [29, 30]. Similarly, tamoxifen has an excitatory effect in the uterus that can increase the risk of cancer in this organ by 3 to 4 times [31]. The increase in brain volume in the tamoxifen and SG 500 + E2V groups is thought to be due to the influence of these hormones on nervous processes; by increasing neurogenesis, training cognition and reduction in the death of neuronal cells [32, 33]. Since the liver, lungs, and kidneys are major detoxification organs of the body [34], a toxicological impairment would be characterized by a decrease or increase in fresh weight compared to normal rats. The significant increase in liver, lung, and spleen wet weights of negative control animals suggests a toxic effect of chronic estradiol exposure. *S. gilo* extract significantly reduced the weights of these organs. This observation highlights the nontoxic appearance of *S. gilo* fruit and justified its use as food for decades. Hematological parameters are markers of toxicity, and a change in the level of one of these parameters beyond physiological limits suggests a metabolic disorder that could be attributed to the presence of a toxicant. The significant increase in the number of circulating leukocytes detected in rats treated with *S. gilo* extract would suggest the infiltration of certain tumors by T lymphocytes and natural killer cells, which has long been considered a sign of favorable prognosis [35], especially in breast cancer [36]. This process is initiated as soon as the tumor cells are detected by the immune system. Tumor development, which is at the expense of healthy tissues, establishes a proinflammatory context through the release of danger signals [37]. Hyperleukocytosis explains the low tumor incidence (17%) due to the anticancer effects of flavonoids, antioxidants, and alkaloids present in *S. gilo* fruits [12, 13, 38, 39]. No metastasis of breast cancer was identified in the femur, brain, and lungs. These results were supported by the histomorphology of the mammary glands of the different experimental groups, where no invasive mammary carcinoma was observed. The most advanced tumor stage was in the negative control with atypical mammary gland hyperplasia. In fact, the alteration sequence of the mammary gland is well known, ranging from terminal ducts to intraductal proliferations (hyperplasia), followed by carcinoma *in situ*, and invasive carcinoma with metastases [40].

## 5. Conclusion

The data presented herein demonstrate that the hydroethanolic extract of *S. gilo* fruits has antiproliferative effects on different breast tumor cells. Moreover, this extract protected female Wistar rats against E2V-induced breast hyperplasia. Furthermore, according to the toxicological profile, this extract appears to be safe. The overall results showed that *S. gilo* extract has potential *in vitro* and *in vivo* antiproliferative properties. This plant may be a potential candidate to improve conventional therapy for breast cancer.

## Figures and Tables

**Table 1:** Quantitative phytochemical analyses and scavenging free radical activities of *S. gilo* hydroethanolic extract.

*Concentration of selected phytochemicals*		

Total proteins	81.97 ± 3.19 mg eq bovine serum albumin	
Total phenols	26.79 ± 0.22 mg eq gallic acid	
Flavonoids	15.94 ± 0.07 mg eq quercetin	
Flavonols	1.59 ± 0.06 mg eq quercetin	

*Radical scavenging activities of S. gilo*		
	EC_50_ (*μ*g/mL)	
	DPPH	ABTS

Control (Ferulic acid)	3.9 ± 0.06	3.7 ± 0.6
*S. gilo*	245.6 ± 8.3	147.1 ± 4.9

EC_50_ = Concentration of *Solanum gilo* extract which results in 50% of scavenging. The results are expressed as mean ± SEM of 3 independent experiments.

**Table 2:** Effects of *S. gilo* hydroethanolic extract on relative wet weight of various organs after 16 weeks of treatment.

Organs	NOR	E2V	TAMOX + E2V	*Solanum gilo* (mg/kg) + E2V		
				125	250	500
Uterus	4817.1 ± 435.2	2981.9 ± 284.9##	864.6 ± 55.7^*∗∗∗*^	2879.2 ± 221.2	2410.7 ± 164.7^*∗*^	2261.1 ± 272.5^*∗∗*^
Liver	25171.2 ± 686.4	29391.3 ± 329.7#	29507.6 ± 1032.6	27970.6 ± 831.2	26873.4 ± 894.3	25253.7 ± 500.4^*∗*^
Lung	7887.3 ± 192.5	8294.7 ± 440.8	8938.1 ± 281.7	7734.6 ± 489.5	8265.7 ± 646.8	6161.2 ± 345.9^*∗*^
Spleen	2640.1 ± 83.5	3488.6 ± 162.5##	2741.9 ± 91.2^*∗∗*^	2621.8 ± 179.8^*∗*^	2902.9 ± 162.3^*∗∗*^	2092.8 ± 102.9^*∗∗∗*^
Adrenals	415.4 ± 8.2	410.1 ± 23.6	318.9 ± 13.9	342.8 ± 22.8	372.8 ± 42.9	287.2 ± 30.3^*∗*^
Kidneys	6474.1 ± 60.3	6276.8 ± 209.9	6808.4 ± 137.2	6001.1 ± 141.5	6026.4 ± 229.4	5629.9 ± 116.1#^*∗*^
Femur	2511.2 ± 63.5	2458.1 ± 38.4	2509.9 ± 38.9	2483.8 ± 91.6	2508.7 ± 73.8	2508.7 ± 73.8
Brain	9543.5 ± 363.1	10215.3 ± 265.6	11304.1 ± 93.1##	10039.5 ± 106.7	10670.6 ± 340.2	11004.7 ± 350.7#
Ovaries	801.4 ± 16.4	735.9 ± 40.6	320.2 ± 40.6^*∗∗∗*^	320.2 ± 13.2^*∗∗∗*^	524.4 ± 23.9^*∗∗*^	499.2 ± 16.1^*∗∗∗*^

NOR = Normal control treated with distilled water; E2V = E2V-control treated with distilled water; SG + E2V = Animals treated with the *S. gilo* hydroethanolic extract at the doses of 125, 250, and 500 mg/kg BW. TAM + E2V = Animals treated with tamoxifen (3.3 mg/kg BW); all groups except the normal group (NOR) received chronic administration of E2V at the dose of 1 mg/kg BW. Data are represented as mean ± SEM (*n* = 6). ^*∗*^*p* < 0.05, ^*∗∗*^*p* < 0.01, and ^*∗∗∗*^*p* < 0.001 as compared to negative control; ##*p* < 0.01 and ###*p* < 0.001 as compared to normal control.

**Table 3:** Effects of *S. gilo* hydroethanolic extract on biochemical parameters after 16 weeks of treatment.

Items	NOR	E2V	TAMOX + E2V	*Solanum gilo* (mg/kg) + E2V		
				125	250	500
Transaminases						
ALT	42.3 ± 2.5	45.8 ± 2.1	45.2 ± 2.2	48.7 ± 4.6	46.2 ± 2.5	39.6 ± 2.8^*∗*^
AST	25.3 ± 3.5	31.2 ± 2.5#	37.7 ± 2.1##	37.8 ± 4.9##	34.6 ± 2.5#	27.1 ± 3.1

Hematological						
WBC (×10^3^ *μ*L^−1^)	14.5 ± 0.7	12.9 ± 0.6	12.9 ± 1.1	10.1 ± 1.5#	8.3 ± 1.1##^*∗*^	6.1 ± 1.1###^*∗∗*^
Lymphocytes (%)	47.5 ± 1.1	57.6 ± 1.6#	46.7 ± 1.1	42.6 ± 1.1	45.5 ± 1.5	41.7 ± 1.3
Monocytes (%)	13.1 ± 0.6	13.1 ± 0.9	19.2 ± 0.9#^*∗*^	20.4 ± 0.7##^*∗∗*^	19.3 ± 1.7#^*∗*^	20.2 ± 2.3#^*∗*^
Granulocytes (%)	39.5 ± 0.6	29.4 ± 0.9#	34.3 ± 1.1	37.2 ± 0.7	35.2 ± 2.7	38.3 ± 1.6
RBC (×10^3^ *μ*L^−1^)	8.5 ± 0.1	8.1 ± 0.1	7.8 ± 0.2	8.4 ± 0.2	8.5 ± 0.3	8.5 ± 0.2
Hematocrit (%)	44.5 ± 0.6	42.7 ± 0.7	42.9 ± 0.5	44.7 ± 0.7	43.2 ± 3.7	48.5 ± 1.5
MCV (fL)	52.3 ± 0.8	52.7 ± 0.3	54.9 ± 0.4	5.6 ± 0.9	59.6 ± 4.2	57.3 ± 0.6
Platelets (×10^3^ *μ*L^−1^)	900.3 ± 32.5	802.6 ± 15.1	789.8 ± 14.5	945.6 ± 25.7	386.6 ± 116.6###^*∗∗∗*^	698.2 ± 63.7
MCH (pg)	16.5 ± 0.2	16.4 ± 0.2	16.9 ± 0.1	16.7 ± 0.3	17.5 ± 0.5	16.9 ± 0.2
Hemoglobin (g/dL)	140.7 ± 2.1	133.6 ± 3.1	133.3 ± 1.8	140.3 ± 2.2	139.2 ± 7.9	144.2 ± 4.5
MCHC (g/dL)	316.3 ± 0.9	312.4 ± 3.2	309.8 ± 1.3	312.4 ± 0.7	331.8 ± 18.1	296.8 ± 2.4

NOR = Normal control treated with distilled water; E2V = E2V-control treated with distilled water; SG + E2V = Animals treated with the *S. gilo* hydroethanolic extract at the doses of 125, 250, and 500 mg/kg BW. TAM + E2V = Animals treated with tamoxifen (3.3 mg/kg BW); all groups except the normal group (NOR) received chronic administration of E2V at the dose of 1 mg/kg BW. Data are represented as mean ± SEM (*n* = 6). ^*∗*^*p* < 0.05, ^*∗∗*^*p* < 0.01, and ^*∗∗∗*^*p* < 0.001 as compared to negative control; #*p* < 0.05; ##*p* < 0.01, ###*p* < 0.001 as compared to normal control.

## Data Availability

The data used to support the findings of this study are included within the article.

## Abbreviations

ALT: Alanine transaminase
AST: Aspartate transaminase
BW: Body weight
DMEM: Dulbecco's Modified Eagle Medium
DMSO: Dimethylsulfoxide
ER: Estrogen receptor
FBS: Fetal bovine serum
HEPES: 4-(2-hydroxyethyl)-1-piperazineethanesulfonic acid
Hb: Hemoglobin
Ht: Hematocrit
MCH: Mean corpuscular hemoglobin
MCHC: Mean corpuscular hemoglobin concentration
MCV: Mean corpuscular volume
NHC: National herbarium of Cameroon
RBC: Red blood cell
RPMI: Roswell Park Memorial Institute medium
SEM: Standard error of mean
TAMOX: Tamoxifen.
